# Evaluation of the Antimicrobial Activity and Cytotoxicity of Different Components of Natural Origin Present in Essential Oils

**DOI:** 10.3390/molecules23061399

**Published:** 2018-06-08

**Authors:** Sara García-Salinas, Hellen Elizondo-Castillo, Manuel Arruebo, Gracia Mendoza, Silvia Irusta

**Affiliations:** 1 Department of Chemical and Environmental Engineering, Aragon Institute of Nanoscience (INA), University of Zaragoza, Campus Río Ebro-Edificio I+D, C/Poeta Mariano Esquillor S/N, 50018 Zaragoza, Spain; saragarciasalinas@gmail.com (S.G.-S.); helizondo02@gmail.com (H.E.-C.); arruebom@unizar.es (M.A.); 2 Networking Research Center on Bioengineering, Biomaterials and Nanomedicine, CIBER-BBN, 28029 Madrid, Spain; 3 Aragon Health Research Institute (IIS Aragón), 50009 Zaragoza, Spain

**Keywords:** antimicrobial, essential oils, monoterpenoids, cytotoxicity, wound dressings

## Abstract

Even though essential oils (EOs) have been used for therapeutic purposes, there is now a renewed interest in the antimicrobial properties of phytochemicals and EOs in particular. Their demonstrated low levels of induction of antimicrobial resistance make them interesting for bactericidal applications, though their complex composition makes it necessary to focus on the study of their main components to identify the most effective ones. Herein, the evaluation of the antimicrobial action of different molecules present in EOs against planktonic and biofilm-forming Gram-positive (*Staphylococcus aureus*) and Gram-negative (*Escherichia coli*) bacteria was assessed. The bactericidal mechanisms of the different molecules, as well as their cytocompatibility, were also studied. Carvacrol, cinnamaldehyde, and thymol exhibit the highest in vitro antimicrobial activities against *E. coli* and *S. aureus*, with membrane disruption the bactericidal mechanism identified. The addition of those compounds (≥0.5 mg/mL) hampers *S. aureus* biofilm formation and partially eliminates preformed biofilms. The subcytotoxic values of the tested EO molecules (0.015–0.090 mg/mL) are lower than the minimum inhibitory and bactericidal concentrations obtained for bacteria (0.2–0.5 mg/mL) but are higher than that obtained for chlorhexidine (0.004 mg/mL), indicating the reduced cytotoxicity of EOs. Therefore, carvacrol, cinnamaldehyde, and thymol are molecules contained in EOs that could be used against *E. coli*– and *S. aureus*–mediated infections without a potential induction of bactericidal resistance and with lower cell toxicity than the conventional widely used chlorhexidine.

## 1. Introduction

Since the discovery of penicillin, the use of antibiotics, originally developed for human healthcare, has been extended to animal therapeutics and agriculture [1]. The use and misuse of antibiotics has led to the emergence of antibiotic resistance in human and animal pathogens, which is recognized as a serious and global concern because resistance in common bacteria has reached alarming levels in all parts of the world [2]. The continued evolution of antimicrobial resistance (AMR) in hospitals is a growing concern because of its potential to endanger the future of antimicrobial drug therapy [3]. Even the new generation of antibiotics is becoming virtually ineffective, and it is predicted that AMR will cause more deaths than cancer-associated diseases by the middle of the century [4]. The discovery of strains resistant to all antibiotics available in the clinic has also made an impact on society in general [5]. Sub-inhibitory antibiotic doses help stepwise selection of resistance, and the resulting resistant clones like methicillin-resistant *Staphylococcus aureus*, *Escherichia coli*, and *Klebsiella* are rapidly disseminated. The overall burden of staphylococcal disease caused by methicillin-resistant *S. aureus* (MRSA) strains is increasing in many countries in both healthcare and community settings [6]. Multiple antimicrobial resistance determinants have been found in *E. coli* on the same plasmid, further facilitating their propagation and co-selection. For instance, the multidrug resistance plasmid IncA/C found in *E. coli*, often encodes for resistance to common antimicrobial agents such as tetracycline, chloramphenicol/florfenicol, streptomycin/spectinomycin, sulfonamides, and extended spectrum β-lactamases, and its spread to pathogenic bacteria may limit antibacterial means to fight infections caused by these bacteria [3].

Because of the emergence of AMR, the Center for Disease Control (CDC) has endorsed the need for the development of new antibiotics. However, the elaboration of new antibiotics is expensive and time-consuming. Meanwhile, the genetic plasticity in pathogens results in the development of resistance at a rapid rate [7]. Therefore, there is a crucial need for research of new substances with the potential to combat resistant strains to minimize their selection. In 2011, academics and industry collaborated on a priority list for approaches to resolve the “antimicrobial-resistance crisis.” Amongst the potential strategies suggested, the development of alternatives to antibiotics were proposed [8]. Compounds from natural sources such as animals, plants, and microorganisms have been highlighted as renewed potential antimicrobial alternatives [9]. Famous seafarers (e.g., Marco Polo) established routes for specie trade, and different compounds of natural origin present in species are still being used today to prevent foodborne pathogens showing low levels of antimicrobial resistance [10]. Antibiotics by definition have a natural origin (i.e., penicillin is derived from *Penicillium fungi*), and antimicrobials of synthetic origin used to fight against infection are considered as drugs (i.e., isoniazid). However, there are several antibiotics that have multiple mechanisms of action. In this regard, essential oils (EO) are oily aromatic substances extracted from plants with antibacterial, antifungal, insecticidal, and antiviral properties. EOs have been distilled for more than 2000 years, and there is now renewed interest in the antimicrobial properties of phytochemicals and EOs in particular. The demonstrated low levels of induction of antimicrobial resistance toward EOs could be related to the fact that these substances do not attack a single specific target but have multiple modes of antibacterial action [1]. Antibiotics, and antiseptics like chlorhexidine, have been shown to be able to generate resistance in *Staphylococcus* [11] by mechanisms (mutations in qacA/B gene) that may be common to other microorganisms. However, EO-based compounds are reported as unable to generate antimicrobial resistances in studies involving Gram negative and Gram positive microorganisms subsequently treated with clove, thyme, cinnamon, and oregano oils [12,13,14,15].

EOs are complex blends of a variety of molecules such as terpenoids, phenol-derived aromatic components, and aliphatic components. Their compositions depend on factors such as seasonal variation, climate, plant organ, age, subspecies, and even the oil extraction method. Consequently, the extracted product can fluctuate in quality, quantity, and composition [16]. Generally, EOs contain about 20–60 components, up to more than 100 single substances, at quite different concentrations; two or three are major components at fairly high concentrations (20–70%) compared to other components that are present only in trace amounts. Because of this, in order to have a systematic evaluation of EOs’ antibacterial activity, it is necessary to focus on the study of their main components.

Different extracted components from EOs such as carvacrol, thymol, eugenol, perillaldehyde, and cinnamaldehyde have been reported as antibacterial agents [17]. However, the reported values for their minimum inhibitory (MICs) and bactericidal (MBCs) concentrations are extremely divergent. For example, the MIC of carvacrol toward *S. aureus* found in the literature ranges from approximately 0.15 mg/mL [18] to 15 mg/mL [19]. In some cases, the different MICs reported could be attributed to the bacteria strain used. Wang et al. [20] reported an MIC value for carvacrol of 0.31 mg/mL for *S. aureus* ATCC 43300, while using *S. aureus* ATCC 6538, Silva da Luz et al. [21] reported a MIC of 2.5 µL/mL (approximately 2.45 mg/mL) for the same component. Even for the same bacteria strain (ATCC 6538), MIC values for carvacrol of 0.4 mg/mL [22] and 0.015% *v*/*v* (approximately 0.147 mg/mL) [18] can be found in the literature.

Beside the well-documented antibacterial action of EO components, there is some evidence corroborating the enhancement in the antimicrobial action of EO components used in combination with other antimicrobial agents, both synthetic and natural [23]. Thymol and carvacrol were found to have additive antibacterial effect against *S. aureus*, *E. coli*, *Salmonella*, and *Bacillus cereus*. Ye et al. [24] tested the synergy between cinnamaldehyde and carvacrol in *S. aureus* and *E. coli* among other bacteria and concluded that cinnamaldehyde and carvacrol exhibit high antibacterial activities and have synergistic antimicrobial action against these bacteria. Thymol and carvacrol were found to give an additive effect when tested against *S. aureus* and *Pseudomonas aeruginosa* [25]. Thymol combined with carvacrol had a synergistic effect against *Salmonella typhimurium* [26].

One interesting application of EO components with antimicrobial activity would be their incorporation into wound dressings since they can prevent or treat wound-associated infections and aid tissue regeneration [27]. Bacterial components have been highlighted as harmful factors during wound healing due to their interference with cell-matrix interactions and due a reduced inflammatory response they produce. In this regard, *S. aureus* colonize from 30% to 50% of healthy adults and is able to rapidly infect skin lesions with a consequential inflammatory process [28]. *E. coli* is also among the main bacterial species that commonly colonize skin wounds, and from this initial colonization, severe problems can occur such as topical infections or even sepsis [29]. Within the clinical settings, biofilm formation is a pressing challenge that leads to chronic infections. Prevention of biofilm formation is considered preferable to its removal, since the latter is a very difficult and demanding task, which can cause recontamination problems due to the uncontrolled release of bacterial cells and toxins after their disruption. One of the outstanding antimicrobial properties of many EOs is that they can also be effective even against microbial biofilms [30].

The aim of this work was to shed light on the evaluation of the bactericidal activity and mechanisms of action of EO-present molecules against *S. aureus* and *E. coli* in planktonic growth. The antibiofilm efficiency and the possibility of synergy between carvacrol (CRV), cinnamaldehyde (CIN) and thymol (THY) were also analyzed. The potential toxicity of those components was also investigated in different cell types, including human dermal fibroblasts, keratinocytes, and macrophages. The main goal of this work is to deepen our understanding of the effects and mechanisms of these purified molecules on bacteria, avoiding the intrinsic variability of their extraction from plants, and focusing on their own ability to hamper bacteria growth and colonization.

## 2. Results

### 2.1. Bactericidal Activity against Planktonic Bacteria

#### 2.1.1. MIC and MBC Values

The antibacterial effect of several components present in different EOs reported as bactericidal, such as carvacrol [31], thymol [32], cinnamaldehyde [33], eugenol [34], β-caryophyllene [35], and rosmarinic acid [36], were studied. Squalene, a well-known natural antioxidant [37], was also included in the study for comparison. Table 1 and Figure S1 show the MIC and MBC results of the components against *E. coli* and *S. aureus*. The most active compounds were THY, CRV, CIN, and eugenol, showing significant differences against the control sample.

#### 2.1.2. Bactericidal Mechanism

**Figure 1:**
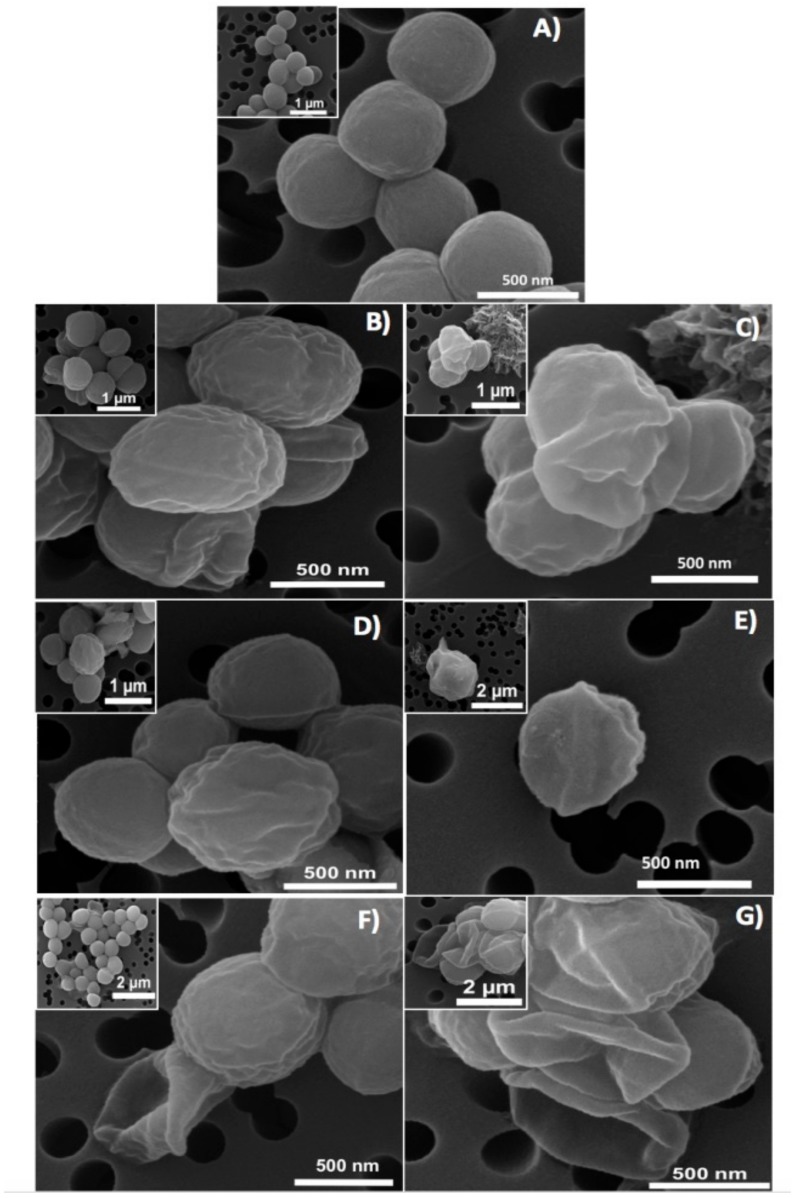
Scanning electron microscopy (SEM) images of *S. aureus* (**A**) untreated (control sample); treated bacteria during 24 h with MIC of (**B**) carvacrol; (**D**) cinnamaldheyde; (**F**) thymol; treated bacteria during 24 h with MBC of (**C**) carvacrol; (**E**) cinnamaldheyde; (**G**) thymol.

**Figure 2:**
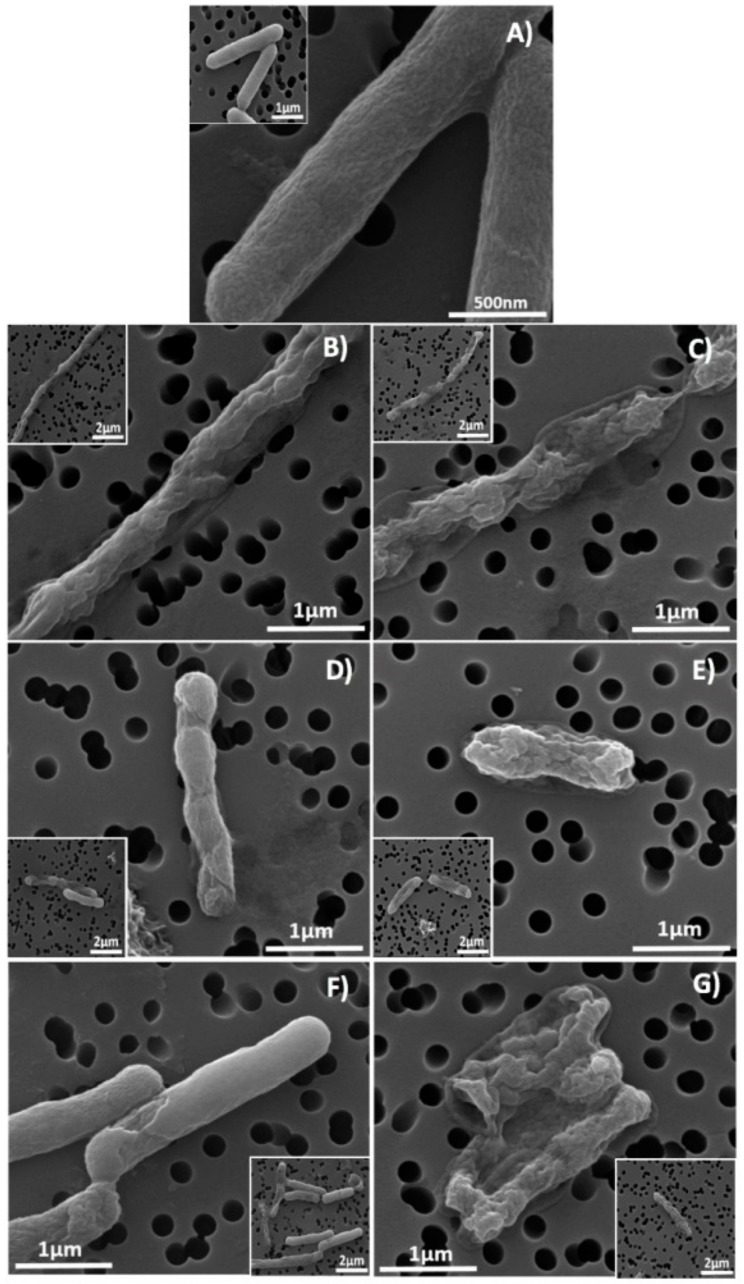
SEM images of *E. coli* (**A**) untreated (control sample); treated bacteria during 24 h with MIC of (**B**) carvacrol; (**D**) cinnamaldheyde; (**F**) thymol; treated bacteria during 24 h with MBC of (**C**) carvacrol; (**E**) cinnamaldheyde; (**G**) thymol.

*E. coli* and *S. aureus* exposed during 24 h to CRV, CIN, and THY at MIC and MBC concentrations were morphologically examined by scanning electron microscopy (SEM) (Figure 1). The morphology and microstructure of *S. aureus* before being exposed to any compound can be observed in Figure 1A, having a normal and spherical shape and a well-preserved cell membrane. However, after exposition to CRV, CIN and THY at MICs for 24 h (Figure 1B–G), the morphology of *S. aureus* was distorted. Part of the cell peptidoglycan structure appeared depressed, indicating an initial damage. *S. aureus* exposed to MBC concentrations during 24 h became deformed and wrinkled, indicating that the intracellular content had leaked out. There was a reduced number of bacteria in the samples, and it was hard to find the ones exposed to CIN, probably due to the severe damage to the bacterial peptidoglycan layer and cell membrane and subsequently cell death and detachment from the filter holder. The reduction in cell size, length, and diameter observed for *S. aureus* in response to the active compound could be reasonably attributed to the leakage of cytosolic fluids outside the cells. *E. coli* untreated cells were rod shaped, regular, and with intact morphology (Figure 2A) in contrast to MIC-treated cells (Figure 2B–G). SEM images showed morphological alterations and lyses of the outer membrane integrity in cells exposed at MICs. At MBC, a complete lysis or seriously damaged cells were observed.

**Figure 3:**
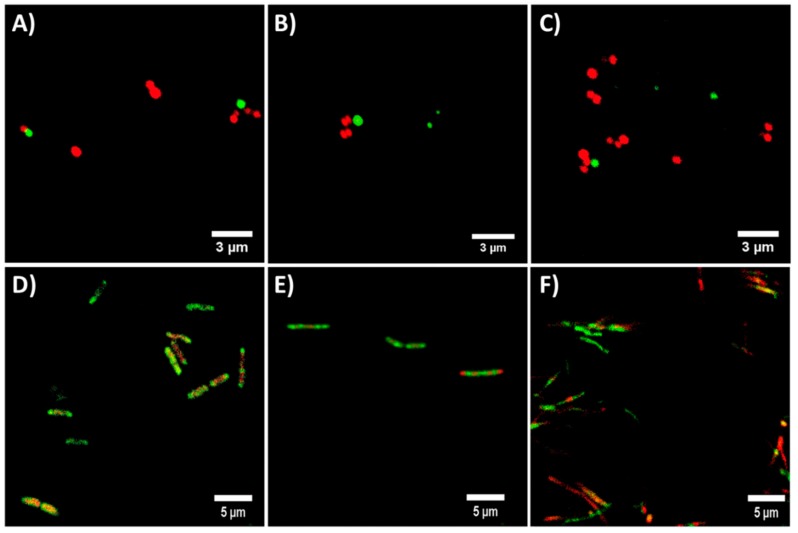
Confocal microscopy images of *S. aureus* (**A**–**C**) and *E. coli* (**D**–**F**) treated with the MIC of carvacrol (**A**,**D**), cinnamaldheyde (**B**,**E**) and thymol (**C**,**F**), stained with the Live/Dead^®^BacLight™ bacterial viability kit. Red staining displays membrane damage.

In order to confirm the bactericidal mechanism of the active compounds present in EOs, flow cytometry and confocal microscopy studies were developed. Flow cytometry histograms (Figure S2) displayed peaks in the range of the negative control (damaged membrane caused by chlorhexidine [38]) when *S. aureus* and *E. coli* were treated with the tested compounds at MBC, which is consistent with cell membrane disruption as previously reported [39]. Only CIN-treated cells show peaks slightly displaced toward the positive control (undamaged membrane) for both microorganisms, suggesting that the involvement of cell membrane disruption in bacteria death was not as clear as the SEM images showed. However, confocal microscopy images (Figure 3 and Figure S3) clearly confirmed membrane damage exerted by the compounds tested when bacteria were incubated with the MICs of the molecules, showing red staining related to membrane integrity compromise. Furthermore, in the case of *E. coli*, the damaged membrane areas can be clearly distinguished (Figure 3D–F). All these results point out to the bacteria membrane disruption as the bactericidal mechanism exerted by the compounds present in the EOs.

#### 2.1.3. Synergism

Synergistic interactions between EO active compounds may increase their efficacy as antibacterial agents. In our studies, the Fractional Inhibitory Concentration Index (FICI) values obtained against *S. aureus* (Table 2; Figure S4) indicate that only the CRV-THY combination has an additive effect, while CIN has no interaction with the other compounds tested. These results may be related to their chemical structure as CRV and THY have almost the same molecular structure (Table 1). It is worth noting that all the FICI values are smaller than 4.0 indicating that there is no antagonism between the tested active compounds.

### 2.2. Antibiofilm Activity

**Figure 4:**
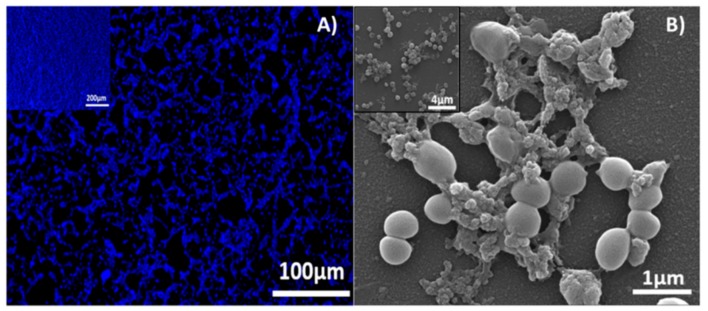
Calcofluor staining (**A**) and SEM images of *S. aureus* biofilm formed after 16 h (**B**).

**Figure 5:**
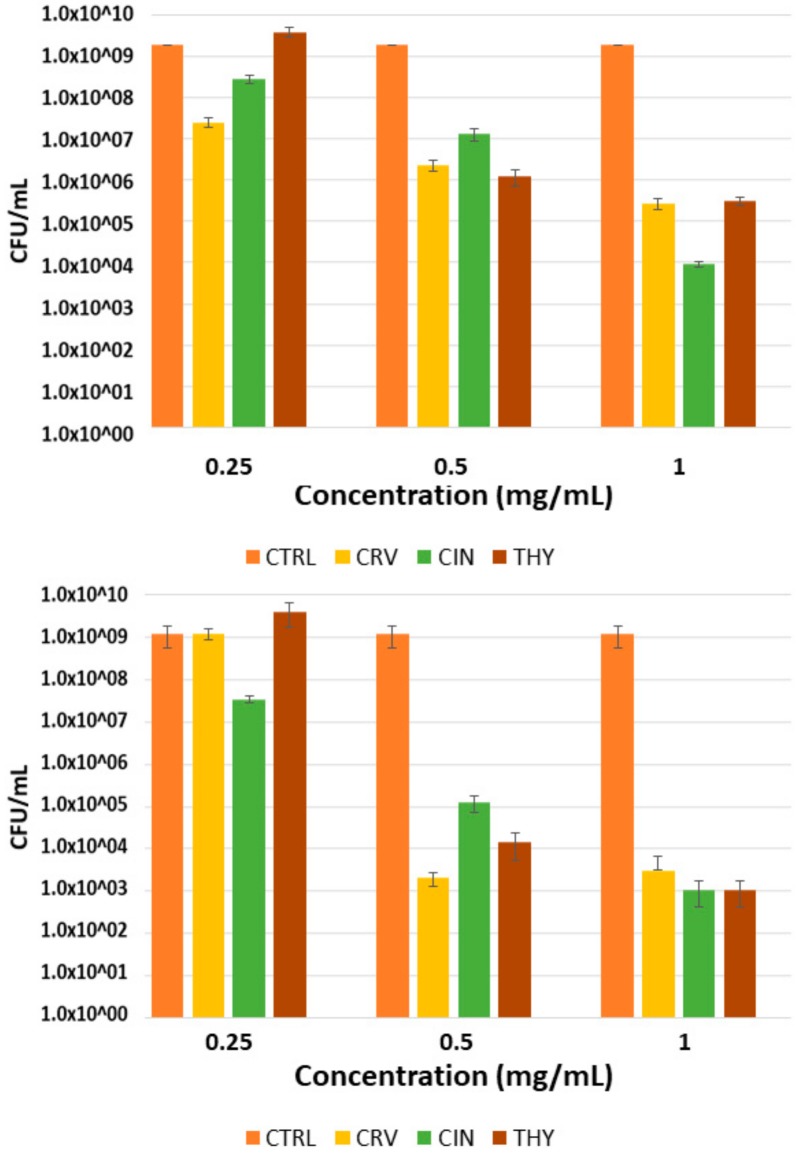
Effect of EOs components at different concentrations on *S. aureus* biofilm: elimination of preformed biofilm (**A**) and inhibition of biofilm formation (**B**). CTRL = Control sample (not treated biofilm), CRV = biofilm treated with Carvacrol, CIN = biofilm treated with cinnamaldehyde, THY = biofilm treated with thymol.

*S. aureus* biofilm formation was observed by calcofluor white staining and by SEM analysis after incubation for 16 h (Figure 4A,B, respectively). The quantification (colony forming units per milliliter = CFU/mL) carried out after incubation of the formed biofilm (Figure 5A) with the antimicrobial compounds has shown a statistically significant decrease in bacteria growth compared to the untreated biofilms. At 0.5 mg/mL of EO-contained molecules, preformed biofilms showed a reduction in bacteria growth around 2 logs when biofilm was treated with CIN, while CRV and THY exerted a superior decrease (3 logs). The highest tested concentration (1 mg/mL) showed a reduction in CFU/mL higher than 5 logs. As expected, concentrations of the active molecules higher than MIC and even MBC values obtained for planktonic bacteria are needed for biofilm elimination.

The addition of these compounds to the bacteria suspension before biofilm formation hindered this process since there was a significant decrease in the posterior bacterial growth (4 logs for CIN) (Figure 5B). THY and CRV produced even higher reductions of about 5 and 6 logs, respectively. Again, the concentrations needed to retard biofilm growth and development were higher than the MIC values retrieved for planktonic bacteria (Table 1).

### 2.3. Cytotoxicity

**Figure 6:**
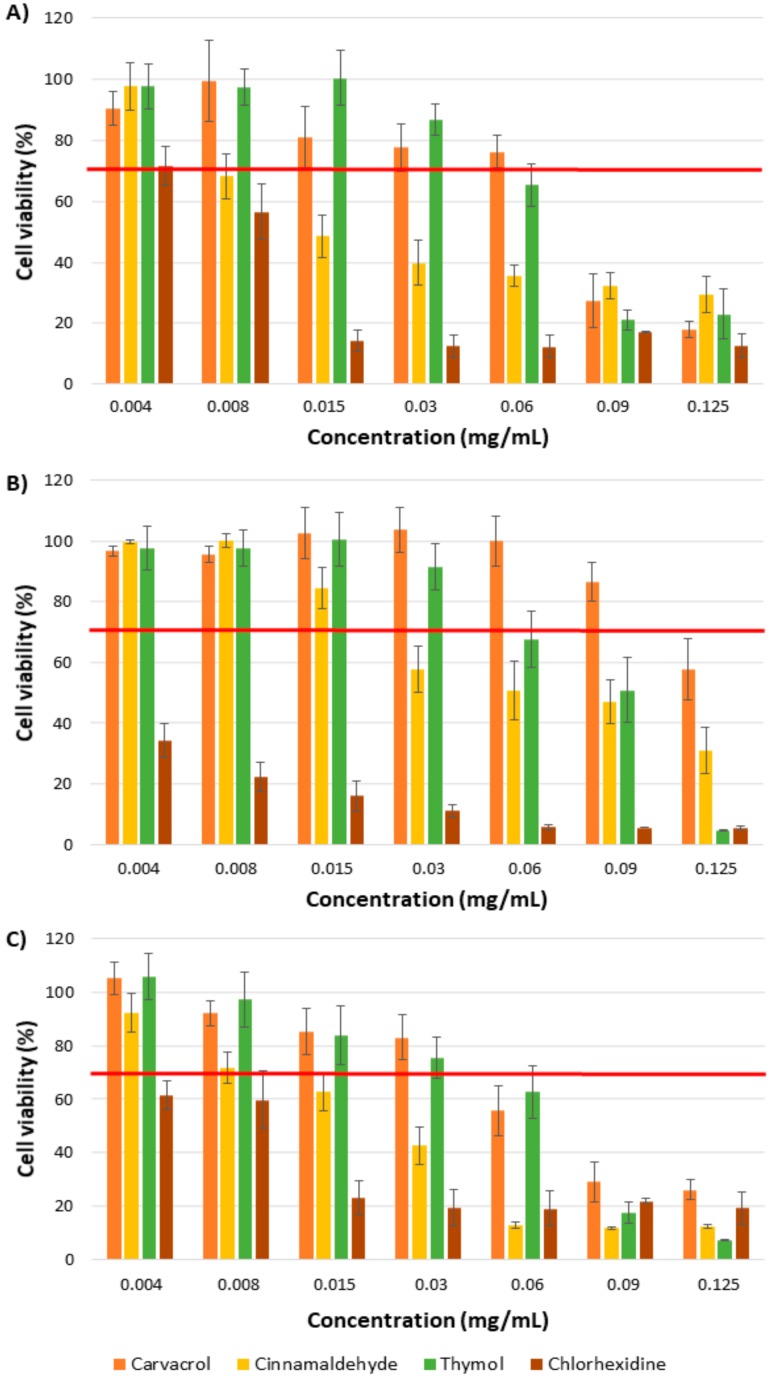
Cell viability after treatment with carvacrol, cinnamaldehyde, thymol and chlorhexidine for 24 h on human dermal fibroblasts (**A**); macrophages (**B**); keratinocytes (**C**). Control sample (untreated cells) = 100% viability.

Cytocompatibility and not cytotoxicity is required for a wound-healing product since it would be in contact with the infected wound tissue and its neighboring eukaryotic cells. The cytotoxicity activities of these antimicrobial compounds were investigated using fibroblasts, macrophages, and keratinocytes cell lines (Figure 6). Inflammatory cells such as macrophages are generated during wound healing [40], whereas keratinocytes and fibroblasts are part of the epidermis and dermis, respectively. Due to the insolubility of those compounds in aqueous media, those were dispersed using Tween^®^ 80 (Sigma-Aldrich, St. Louis, MO, USA) as described in the materials and methods section. Hence, the cytotoxicity of the free compounds would be reduced in absence of Tween^®^ 80 due to their nonpolar character.

CIN was the most cytotoxic chemical of the tested molecules; a dose of 0.030 mg/mL of this compound was enough to reduce the viability of keratinocytes and macrophages below 70% (lowest value established by the ISO 10993-5 [41] to consider a material as non-cytotoxic), and 0.015 mg/mL affects the fibroblasts viability (Figure 6). THY and CRV can be considered toxic to fibroblasts at concentrations equal or higher than 0.090 mg/mL, and the calculated subcytotoxic doses for keratinocytes were 0.060 and 0.030 mg/mL, respectively. There is also a difference between the effect of THY and CRV on macrophages, since subcytotoxic concentrations were 0.060 and 0.090 mg/mL, respectively.

Chlorhexidine, a typical disinfectant and antiseptic drug used in skin disinfection, was tested for comparison. For the studied concentrations (0.004–0.125 mg/mL), chlorhexidine reduces the viability of the three cellular types to 70% or below. Only fibroblasts show viability higher than 70% in presence of chlorohexidine at 0.004 mg/mL. For keratinocytes and macrophages, the subcytotoxic concentration was lower than 0.004 mg/mL.

These subcytotoxic values of the tested compounds were lower than the MICs and MBCs retrieved for bacteria but higher than those obtained with chlorhexidine. In order to reduce bacterial burden in wounds, topical antiseptic agents, among them chlorohexidine gluconate, are usually applied as 2 and 4 *v*/*v* % topical solutions, concentrations five orders of magnitude higher than the subcytotoxic doses. Therefore, in our study, the presence of antibacterial compounds of natural origin in a wound dressing material at MBC concentrations would be only three orders of magnitude higher than the subcytotoxic dose in the worst case scenario demonstrating that those natural origin compounds are less harmful against eukaryotic cells than conventional antiseptics.

## 3. Discussion

Microorganism resistance to antibiotics and antiseptics has become a serious problem in the treatment of infections and results in the imperative search of novel antibacterial approaches. In this regard, EO-based compounds have been pointed out as a suitable strategy due to their bactericidal properties together with their inability to generate antimicrobial resistances [12,13,14,15]. Previous studies have highlighted these promising attributes [17,18,19,20,21,22] though their complex composition is tightly joined to different factors (i.e., seasonal variation, age), which means variability in their bactericidal effects. Thus, in order to delve into EOs antibacterial activity, it is necessary to focus on the study of their main components.

Our work focuses on the bactericidal effects and the mechanisms of action of purified EO-present molecules (CRV, CIN, THY), circumventing their intrinsic variability associated to their plant extraction, against Gram positive (*S. aureus*) and Gram negative (*E. coli*) bacteria in order to elucidate their own ability to kill bacteria. MIC and MBC studies (Table 1 and Figure S1) pointed to THY, CRV, CIN and eugenol as the most effective studied molecules against *E. coli* and *S. aureus* showing the lowest concentrations to inhibit or hamper planktonic bacteria growth. The bactericidal mechanism of the most effective EO-derived molecules (CRV, THY, CIN) was assessed by SEM, flow cytometry and confocal microscopy after treatment of *E. coli* and *S. aureus* planktonic cultures at MIC and MBC concentrations for 24 h (Figure 1, Figure 2 and Figure 3, Figures S2 and S3). These methodologies pointed to membrane disruption as the bactericidal mechanism exerted by these molecules. It is known that phenols, terpenes and aldehydes antibacterial effect is due to their action against the cell cytoplasmic membrane [42]. It has been reported that CRV and THY disturb the membrane integrity, increasing the membrane permeability and causing a leakage of protons and potassium finally leading to the loss of membrane potential [43]. Di Pascua et al. [42] suggested that the presence of the hydroxyl group in CAR and THY is related to the inactivation of the microbial enzymes. This group would interact with the cell membrane causing leakage of cellular components, a change in fatty acids and phospholipids, and an impairment of the energy metabolism influencing genetic material synthesis. However, some authors have pointed out to different bactericidal mechanisms of action for both compounds due to the different location of the hydroxyl group in their structure affecting cell membrane permeability [44], while others agree with our results, showing similar effects for both compounds on bacterial membrane structure [45]. It is important to point out that the bactericidal action cannot be related only to the OH group since eugenol having also a hydroxyl group exhibited lower bactericidal effect (Table 1). According to the literature, the antibacterial mechanism of CIN is not clear. On one hand, its antimicrobial action was attributed to the inhibition of the amino acid decarboxylase activity to bind proteins and no disintegration of the membrane was observed [24]. However, Nazzaro et al. [42] sustained that like CRV, CIN inhibits the generation of adenosine triphosphate from dextrose and disrupts the cell membrane. In addition, the hydrophobicity of the components present in EOs enables their accumulation in cell membranes disturbing their structures and causing an increase in the permeability allowing intracellular constituents leakage [43]. Our studies would indicate similar trends in MIC and MBC values for CRV, THY, and CIN, as well as the disruption of the bacterial surface as target for their activity by three different experimental techniques analyzed.

On the other hand, synergism between EO active molecules has been shown as more efficient as bactericidal agents. For instance, polyethylene films containing a mixture of CRV and THY entrapped within halloysite nanotubes exhibited superior antimicrobial activity against *E. coli* than films containing the individual compounds alone [44]. The combination of CIN and CRV showed better bactericidal effect compared with the components alone against food-borne bacteria [24]. Zhou et al. [26] reported that CIN had a synergistic effect when combined with THY or CRV against *Salmonella typhimurium*. However, in our case, FICI data obtained against *S. aureus* (Table 2 and Figure S4) pointed to CRV-THY as the most efficient combination displaying an additive effect, while CIN did not exert any synergism. The synergistic mechanism between CIN with CRV or with THY was proposed to be caused by the increase in the membrane permeability that enables CIN to be transported into the cell [24]. But according to the treated bacteria SEM micrographs (Figure 1 and Figure 2), flow cytometry histograms (Figure S2) and confocal microscopy images (Figure 3 and Figure S3), the effect of the three compounds against *E. coli* is mainly outer membrane disintegration and the morphology of treated *S. aureus* was similar for the three active compounds. THY and CRV were previously found to give an additive antimicrobial effect on *S. aureus* [25], which is in accordance with its similar chemical structure (Table 1).

*S. aureus* is involved in a wide range of infections that are difficult to treat because, beside the frequent occurrence of antimicrobial-resistant strains, *S. aureus* often resides within biofilms at the infection site [45]. Biofilms are communities of microorganisms living at an interphase where they attach to each other through the extracellular polymeric substance also known as the biofilm matrix composed of extracellular DNA, proteins, and polysaccharides. Due to the protection of this matrix, bacteria show up to 1000 times greater tolerance to antibiotics and biocides than their planktonic counterparts [46]. Because of this, it is important to find compounds that interfere with the early steps of biofilm formation and slow down its formation rate. As expected, concentrations of the active molecules higher than MIC and even MBC values obtained for planktonic bacteria are needed for biofilm elimination. Our study shows that concentrations higher than 1 mg/mL of any of the compounds tested would be necessary for the total elimination of preformed biofilms. Again, the concentrations needed to retard biofilm growth and development were higher than the MIC values retrieved for planktonic bacteria (Table 1), but they were in the same range than those reported in the literature [32].

Regarding the use of these EO-present molecules against bactericidal infections, i.e., chronic wounds, the evaluation of their cytocompatibility in human cell cultures is advisable. Our study showed subcytotoxic concentrations between 0.015 and 0.090 mg/mL, pointing to CIN as the most cytotoxic molecule assayed in human dermal fibroblasts, keratinocytes, and macrophages. However, the widely used antiseptic chlorhexidine was found more cytotoxic than the EO-based compounds, displaying a subcytotoxic concentration of 0.004 mg/mL. Previous studies have also evaluated the toxicity of different compounds present in EOs on different human cell types, such as fibroblasts [47], intestinal cells [48] or different tumor cell lines [49,50]. Their results show subcytotoxic concentrations for CRV and THY in the same range as ours (~500 µM) [48] or higher (50% viability at ~5 µg/mL) [47] and also very similar for CIN (~10 µg/mL) [50] pointing to apoptosis and membrane damage as key cytotoxic mechanisms. Even though the studied molecules showed cytotoxic activity at doses above 0.06–0.09 mg/mL, it is important to point out that during the regenerative process in an infected wound the antimicrobial compound at those doses would eradicate both bacteria and eukaryotic cells, but while bacteria are removed from the injury, eukaryotic cells are continuously arriving to the wound to participate in the regenerative process [27]. Hence, only a small fraction of eukaryotic cells would be damaged.

Finally, the present work has shown the bactericidal effects and mechanisms of promising antibacterial purified EO-based compounds (CRV, THY, CIN), avoiding the variability of plant extracted compounds, for their incorporation to different clinical treatments, i.e., wound dressings for chronic wounds. Their efficiency against both planktonic and biofilm bacteria highlights their potential to be included in bactericidal approaches in order to develop novel strategies against antibiotic and antiseptic resistance.

## 4. Materials and Methods

### 4.1. Materials

Carvacrol (CRV), cinnamaldehyde (CIN), thymol (THY), Squalene, Rosmarinic acid, β-Caryophyllene, Calcofluor White Stain, phorbol 12-myristate 13-acetate (PMA), and Tween^®^ 80 were purchased from Sigma-Aldrich (St. Louis, MO, USA), while Eugenol was supplied by Acros Organics (Gell, Belgium). Tryptone soy broth (TSB) and agar (TSA) were obtained from Conda-Pronadisa (Madrid, Spain) and *S. aureus* (ATCC 25923) from Ielab (Alicante, Spain). Regarding cell lines, human dermal fibroblasts were purchased from Lonza (Bornem, Belgium), and THP1 human monocytes (ATCC TIB-202) from LGC Standards (Barcelona, Spain), while human keratinocytes were kindly gifted by Dr Pilar Martín-Duque. High-glucose DMEM (DMEM w/stable glutamine), RPMI 1640 w/stable glutamine, and antibiotic-antimycotic (60 µg/mL penicillin, 100 µg/mL streptomycin and 0.25 µg/mL amphotericin B) were supplied by Biowest (CEDEX, France). Cell culture reagents, such as fetal bovine serum (FBS), HEPES, nonessential amino acids, 2-mercaptoethanol 50 mM and sodium pyruvate 100 mM, were obtained from Gibco (Manchester, UK), and the Blue Cell Viability assay from Abnova (Aachen, Germany).

### 4.2. Bacteria Culture

A Gram-negative model *E. coli* S17 strain was used, which was kindly donated by Dr. Jose Antonio Ainsa, exerting resistance to streptomycin as it is also widely used for transformation purposes [51]. *S. aureus* ATCC 25923, well-known as not a resistant strain and used in susceptibility tests [52,53], was evaluated as a Gram-positive model. Both strains were initially grown overnight in TSB at 37 °C under shaking (150 rpm) obtaining, in the stationary growth phase, 10^8^–10^9^ CFU/mL. TSA was used for seeding bacteria in an incubator at 37 °C (Memmert, Schwabach, Germany) in order to calculate the bacteria growth (CFU/mL) after treatment with the EO-contained molecules.

### 4.3. Biofilm Formation

*S. aureus* was grown overnight in TSB until stationary growth phase was reached. At this point, bacteria were adjusted to 10^7^ CFU/mL and added to a MW96 microplate and incubated at 37 °C for 16 h without shaking. After incubation, culture medium was discarded and biofilms were washed twice with PBS. In order to determine biofilm formation, Calcofluor White Stain (50 µL) was added to each well and incubated 1 min in the dark at room temperature. After incubation, the stain was removed and biofilms were washed twice with PBS. Samples were air-dried in the dark to be further visualized in an inverted fluorescence microscope (Olympus IX81).

To be analyzed by SEM, biofilms were grown on sterile glass slides incubated in a *S. aureus* planktonic suspension (10^7^ CFU/mL) at 37 °C for 16 h without shaking. Then, biofilms were washed twice with PBS (0.1 M) and fixed in 2.5% glutaraldehyde for 3 h. Samples were dehydrated through a series of ethanol solutions (30, 50, 70, 80, 90 and 100%; 15 min, twice). Finally, samples were air-dried at room temperature and coated with Pt to allow electronic observation. SEM images were acquired in the energy range of 10–15 keV in an SEM Inspect™ F50 (FEI Co., Hillsboro, OR, USA).

### 4.4. Antibacterial Activity

#### 4.4.1. MIC and MBC Determination

Inhibitory and bactericidal concentrations of active EO molecules were tested in two bacteria cultures, *E. coli* and *S. aureus*, following the broth microdilution method. Liquid growth medium containing an inoculum of 10^5^ CFU/mL and serial concentrations of the EO compounds (0.1–4 mg/mL) were used. EO compounds were solubilized in culture medium by adding Tween^®^ 80 (1.5–2% *v*/*v*) prior to their serial dilution. Once bacteria suspension was in stationary growth (10^8^–10^9^ CFU/mL), it was further diluted to ~10^5^ CFU/mL and added to different concentrations (0.01–4 mg/mL) of the antimicrobial agents. Then, samples were incubated for 24 h at 37 °C under shaking (150 rpm). After incubation, bacterial suspensions were diluted in PBS and spot-plated on TSA plates to count colonies after incubation at 37 °C for 24 h. Positive control (untreated bacteria) and negative control (chlorhexidine treated bacteria) samples were also tested.

#### 4.4.2. SEM

Bacteria morphology before and after treatment with EO molecules was analyzed by SEM as we previously reported [54]. Briefly, logarithmic growth phase *E. coli* and *S. aureus* bacteria cultures (~10^5^ CFU/mL) were treated with the selected EO compounds at MIC and MBC values and incubated overnight at 37 °C. Following incubation, samples were spin-dried at 600 g and washed twice in PBS (0.1 M). Bacteria were fixed in 2.5% glutaraldehyde for 90 min and subsequently filtered and dehydrated in ethanol solutions series (30, 50, 70, 80, 90, and 100%; twice for 15 min). Finally, samples were air-dried at room temperature and covered with Pt. SEM micrographs were acquired in a SEM Inspect F50 equipment (FEI Co., LMA-INA, Zaragoza, Spain).

#### 4.4.3. Flow Cytometry

In order to study the bactericidal mechanism of the different molecules present in EOs, *E. coli* and *S. aureus* bacteria samples (10^7^ CFU/mL) were centrifuged at 4400× *g* for 10 min and resuspended in the different compound solutions at MIC and MBC concentrations following the protocols previously described [39,54]. Control groups (not treated and chlorhexidine treated bacteria) were also analyzed. All samples were incubated overnight at 37 °C. After propidium iodide (25 µg/mL; Sigma-Aldrich, Munich, Germany) addition, samples were analyzed by flow cytometry in Gallios equipment (Beckman Coulter Company, Cell Separation and Cytometry Unit, CIBA, IIS Aragon, Zaragoza, Spain).

#### 4.4.4. Confocal Microscopy

The Live/Dead^®^BacLight™ bacterial viability kit (Molecular Probes, Fisher Scientific, Hampton, USA) was used to detect bacteria membrane damage. The methodology is based on the double-staining by SYTO9 and propidium iodide as indicated by the manufacturer. Bacteria samples incubated at 24 h (10^7^ CFU/mL) and treated with the selected EO compounds at MIC were washed in sterile saline solution and further put in contact with the dye mixture for 15 min in the dark at room temperature. Samples were then mounted on slides and visualized by confocal microscopy (Leica TCS SP2 Laser Scanning Confocal Microscope, Microscopy Unit, CIBA, IIS Aragon, Zaragoza, Spain). Control samples were also tested as described above.

#### 4.4.5. Synergy Studies

The Broth Dilution Checkerboard test was used to evaluate the interaction among the three most promising bactericidal EO molecules determined by the MIC and MBC studies against *S. aureus* as previously reported [55]. In brief, a solution containing four times the MBC of each compound was prepared. By using a MW96 plate and fresh medium, compound A was diluted two-fold in vertical orientation and compound B was diluted two-fold in horizontal direction. Then, a bacterial suspension (10^6^ CFU/mL, 100 µL) was added and the plate was incubated overnight at 37 °C. After incubation, bacteria growth was determined by the resazurin assay. The Fractional Inhibitory Concentration Index (FICI) of the combination of compounds A and B was calculated according to the following equation:

$$FICI\;=\;{\mathrm{FIC}}_{A}+{\mathrm{FIC}}_{B}$$

where

$${\mathrm{FIC}}_{A}\;=\;\frac{MICA\;in\;presence\;of\;B}{MIC\;of\;A}\;\mathrm{and}\;{\mathrm{FIC}}_{B}\;=\;\frac{MICB\;in\;presence\;of\;A}{MIC\;of\;B}$$

*FICI* results were classified as synergy (*FICI* < 0.5), addition (0.5 ≤ *FICI* ≤ 1), indifference (1 < *FICI* ≤ 4) or antagonism (*FICI* > 4), as previously described [56].

#### 4.4.6. Biofilm Disruption

The effects of EO molecules (0.25–1 mg/mL) to prevent the formation of biofilm and to disrupt an already formed *S. aureus* biofilm were studied. EO compounds were added to preformed biofilms and samples were incubated for 24 h at 37 °C without shaking. After incubation, biofilms were disrupted by sonication (15 min, 200 W; Ultrasons, JP Selecta, Barcelona, Spain). Samples were then diluted and seeded onto agar plates to count the viable colonies grown after 24 h of incubation at 37 °C.

To study the effects of the compounds present in the EOs on biofilm formation, those were added to bacterial suspensions (10^7^ CFU/mL) in a MW96 microplate and incubated for 16 h at 37 °C without shaking. After incubation, planktonic cells were removed by washing them twice with PBS. Biofilm samples were then sonicated as described above and serially diluted to be further plated on agar. Viable bacteria (CFU/mL) were counted after 24 h of incubation at 37 °C.

#### 4.4.7. Cell Culture and Cytotoxicity Assays

Human dermal fibroblasts, human epidermal keratinocytes (HaCaT), and THP1 human monocytes were used to evaluate the cytotoxic effects of EO-based compounds.

Fibroblasts and HaCaT were routinely grown in high-glucose DMEM supplemented with 10% FBS and antibiotic-antimycotic. Monocytes were cultured in RPMI 1640 supplemented with 10% FBS, 1% HEPES, 1% nonessential amino acids, 0.1% 2-mercaptoethanol 50 mM, 1% sodium pyruvate 100 mM, and antibiotic-antimycotic. Macrophages were obtained by the in vitro differentiation of monocytes by adding 1 µM PMA to the cell culture. All cell types were grown in a humidified atmosphere at 37 °C and 5% CO_2_.

The cytotoxicity was determined by measuring cell metabolism through the Blue Cell Viability assay. Cells were seeded on MW96 microplates and incubated with the tested molecules (0.004–0.125 mg/mL) for 24 h. Control samples (not treated and chlorhexidine treated) were also analyzed. Then, the reagent was added (10%) and cells were incubated for 4 h at 37 °C. The reduction of the dye by metabolically active cells was monitored in a microplate reader (Multimode Synergy HT Microplate Reader; Biotek, Winooski, WI, USA) at 535/590 nm ex/em. Cell viability was determined by interpolation of the emission data obtained from the treated samples and the control samples (not treated cells, 100% viability).

#### 4.4.8. Statistical Analysis

Results are reported as mean ± SD. The normal distribution of the variables was analyzed by the Shapiro-Wilk test followed by the U-Mann-Whitney or Student test (StataSE 12 statistical software, StataCorp LP, Texas, TX, USA). Statistically significant differences among groups were considered when *p* ≤ 0.05.

## 5. Conclusions

Compounds present in EOs including CRV, CIN, and THY exhibit the highest in vitro antimicrobial activities against *E. coli* and *S. aureus* of all the antimicrobials tested. THY showed the lowest MBC values (0.3 mg/mL) among all of the compounds tested and was the most effective bactericide against the Gram-negative and Gram-positive strains evaluated. According to SEM images, flow cytometry and confocal microscopy bacteria membrane disruption is the bactericidal mechanism attributable to CRV, CIN, and THY. There was no antagonism between the tested active compounds, but no synergism was found either; only the CRV-THY combination showed an additive effect. The presence of those compounds at concentrations above 0.5 mg/mL hinders *S. aureus* biofilm formation and also partially eliminates preformed biofilms. The subcytotoxic values of the tested EO compounds (0.015–0.090 mg/mL) are lower than MICs and MBCs for bacteria but much higher than chlorhexidine doses (0.004 mg/mL). The presence of those antibacterial compounds at MBC concentrations would be only three orders of magnitude higher than the subcytotoxic dose in the worst-case scenario.

## Figures and Tables

**Table 1:** Chemical structure and minimal inhibitory concentration (MIC) and minimal bactericidal concentration (MBC) values of different essential oil contained compounds. Average of 12 replicas each compound.

Active Compound	Structure	MIC (mg/mL)		MBC (mg/mL)	
		*E. coli*	*S. aureus*	*E. coli*	*S. aureus*
Carvacrol	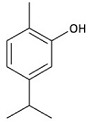	0.2	0.2	0.4	0.3
Cinnamaldehyde	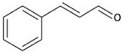	0.2	0.4	0.3	0.5
Thymol	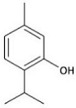	0.2	0.2	0.3	0.3
Eugenol	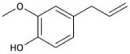	0.4	1.3	0.5	1.5
β-caryophyllene	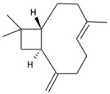	>4.0	>4.0	>4.0	>4.0
Rosmarinic acid	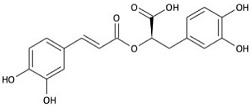	>4.0	2.5	>4.0	4.0
Squalene	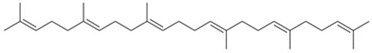	>4.0	>4.0	>4.0	>4.0

**Table 2:** Fractional Inhibitory Concentration (FIC) and Fractional Inhibitory Concentration Index (FICI) values for active compounds combination.

Carvacrol-Cinnamaldehyde				Carvacrol-Thymol				Thymol-Cinnamaldehyde			
FIC_CRV_	FIC_CIN_	FICI		FIC_CRV_	FIC_THY_	FICI		FIC_THY_	FIC_CIN_	FICI	
0.7	1.0	1.7	NI ^1^	0.4	0.5	0.9	ADD ^2^	0.8	0.4	1.2	NI ^1^

^1^ NI: No interaction. ^2^ ADD: Additive.

## Supplementary Materials

The following are available online at http://www.mdpi.com/1420-3049/23/6/1399/s1, Figure S1: Bacteria growth (CFU/mL) for *E. coli* and *S. aureus* at MIC values for carvacrol, cinnamaldehyde and thymol active compounds, Figure S2: Flow cytometry histograms at MBC on *S. aureus* and *E. coli*, Figure S3: *S. aureus* and *E. coli* confocal images after treatment with carvacrol, cinnamaldehyde and thymol, Figure S4: Synergistic effects of the EO molecules assayed against *S. aureus*.
