# Supplementary material for: Evaluation of the Antimicrobial Activity and Cytotoxicity of Different Components of Natural Origin Present in Essential Oils
Source: Molecules. 2018 Jun 8;23(6):1399. doi: 10.3390/molecules23061399 (PMC6100501; doi:10.3390/molecules23061399)
Supplement: Supplementary file 1 [file molecules-23-01399-s001.pdf]

Supplementary material

Evaluation of the Antimicrobial Activity and Cytotoxicity of Different Components of Natural Origin Present in Essential Oils

Sara García-Salinas [1,†], Hellen Elizondo-Castillo [1,†], Manuel Arruebo [1,2,3], Gracia Mendoza [1,3,*] and Silvia Irusta [1,2,3,*]

[1] Department of Chemical and Environmental Engineering, Aragon Institute of Nanoscience (INA), University of Zaragoza, Campus Río Ebro-Edificio I+D, C/Poeta Mariano Esquillor S/N, 50018 Zaragoza, Spain; saragarciasalinas@gmail.com (S.G.-S.); helizondo02@gmail.com (H.E.); arruebom@unizar.es (M.A.)

[2] Networking Research Center on Bioengineering, Biomaterials and Nanomedicine, CIBER-BBN, 28029 Madrid, Spain

[3] Aragon Health Research Institute (IIS Aragón), 50009 Zaragoza, Spain

* Correspondence: gmmenc@unizar.es (G.M.); sirusta@unizar.es (S.I.); Tel.: +34-876-555-437 (S.I.)

† These authors contributed equally to this work.

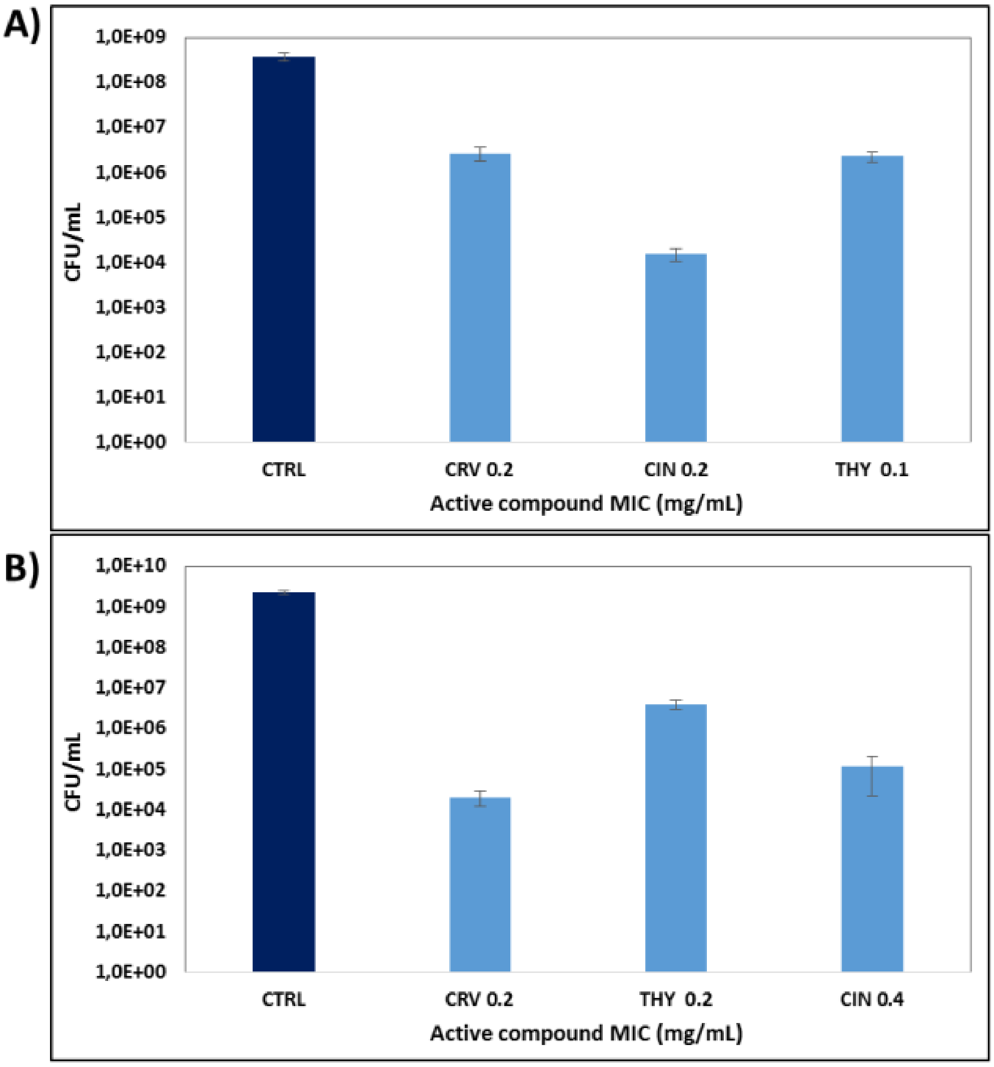

Figure S1. Bacteria growth (CFU/mL) for *E. coli* (**a**) and *S. aureus* (**b**) at MIC values for Carvacrol (CRV), Cinnamaldehyde (CIN) and Thymol (THY) active compounds.

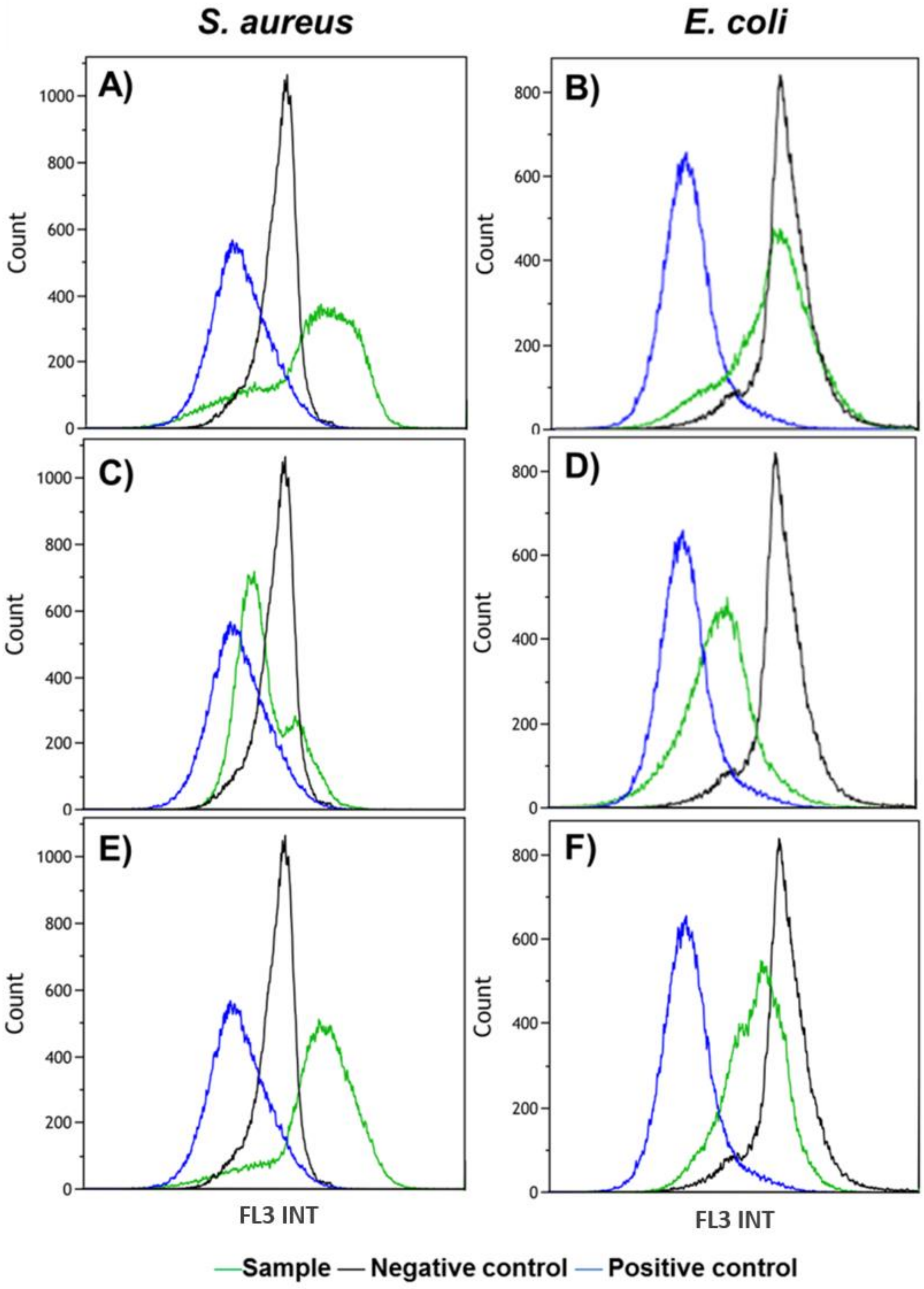

Figure S2. Flow cytometry histograms at MBC on *S. aureus* and *E.coli* of carvacrol (**a**,**b**), cinnamaldehyde (**c**,**d**) and thymol (**e**,**f**). Control samples are also depicted, not treated bacteria (positive control) and bacteria treated with chlorhexidine (negative control), showing alive (intact cell membrane) and dead cells (cell membrane disrupted), respectively.

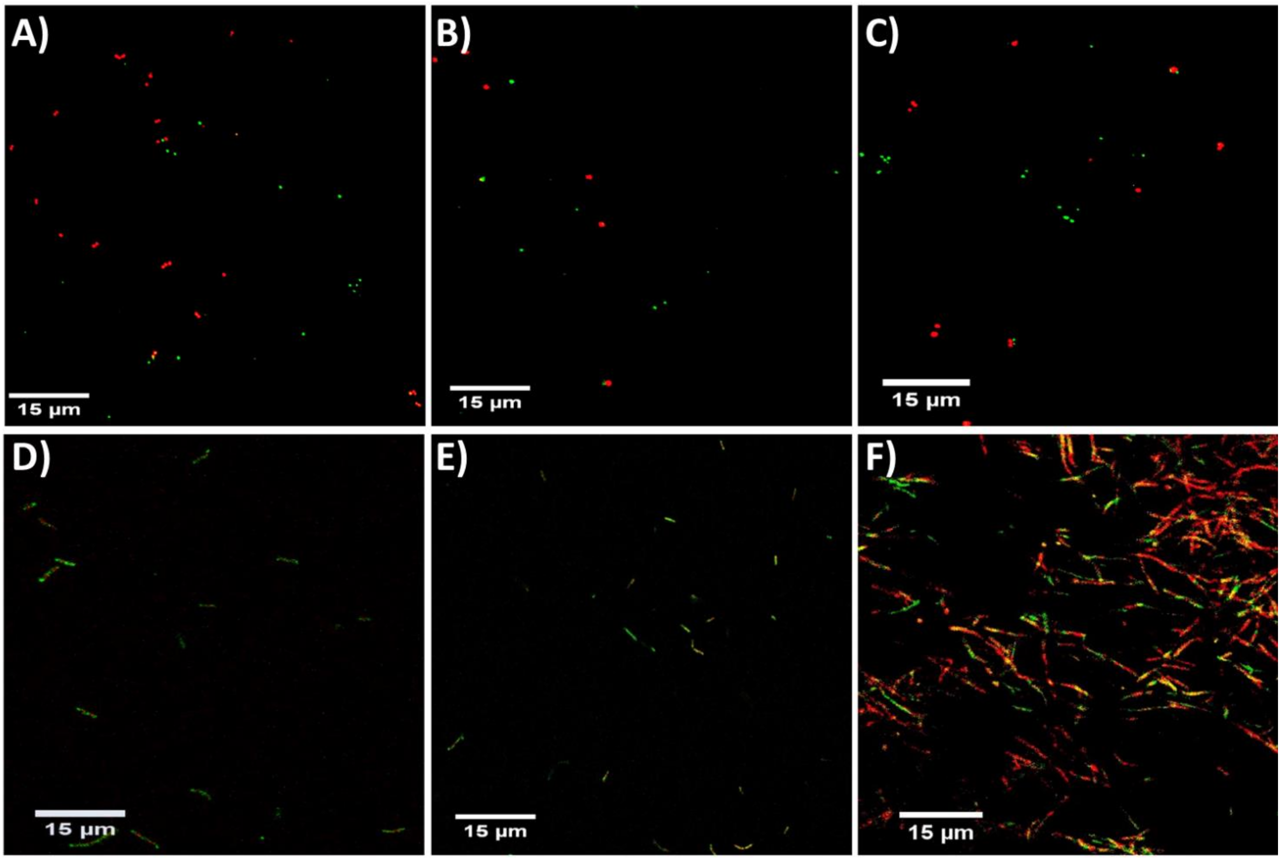

Figure S3. *S. aureus* (**a-c**) and *E. coli* (**d-f**) confocal images after treatment with carvacrol (**a,d**), cinnamaldheyde (**b,e**) and thymol (**c,f**). Red staining displays membrane damage.

	CRV-CIN	CRV-THY	THY-CIN
FICI	1.7±0.5	0.7±0.1	1.5±0.5
Effect	No interaction	Additive	No interaction

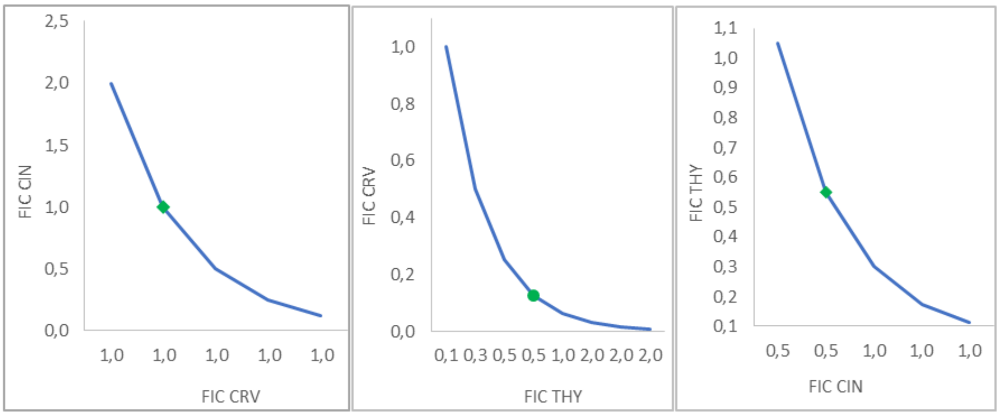

Figure S4. Synergistic effects of the EO molecules assayed (CRV=Carvacrol, CIN=Cinnamaldehyde, THY=Thymol) against *S. aureus.* The Fractional Inhibitory Concentration Index (FICI) for all combinations of EO compounds is shown. The charts depict the Fractional Inhibitory Concentrations (FIC) and the FICI for each combination of compounds.
